# Eye Safety of Light-Emitting Diodes in Toys: The Results of Three Years of Market Surveillance

**DOI:** 10.3390/ijerph23070880

**Published:** 2026-07-06

**Authors:** Dina Attia, Olivier Enouf, Francine Behar-Cohen, Christophe Martinsons

**Affiliations:** 1 Unité d’Evaluation des Risques des Agents Physiques, Département de l’Evaluation des Risques, Agence Nationale de Sécurité Sanitaire de l’Alimentation, de l’Environnement et du Travail (ANSES), 94700 Maisons-Alfort, France; dina.attia@anses.fr; 2 Pôle Photonique et Energétique, Laboratoire National de Métrologie et d’Essais (LNE), 78190 Trappes, France; olivier.enouf@lne.fr; 3 Physiopathologie des Maladies Oculaires, Centre de Recherche des Cordeliers, Sorbonne Université, INSERM UMR S1138, Université Paris Cité, 75006 Paris, France; francine.behar@gmail.com; 4 OphtalmoPôle, Cochin Hospital, Assistance Publique—Hôpitaux de Paris, 75014 Paris, France; 5 Direction Santé, Ambiances et Mobilité, Centre Scientifique et Technique du Bâtiment (CSTB), 38400 Saint Martin d’Hères, France

**Keywords:** LED, toys, photobiological safety, retina, glare, blue-light hazard, thermal hazard, market surveillance, eye, safety, toy, vision, visual health

## Abstract

**Highlights:**

**Public health relevance—How does this work relate to a public health issue?**

- Light-emitting diodes (LEDs) have become ubiquitous in toys, exhibiting a wide range of functions ranging from use as low-intensity indicators to high-intensity lighting devices.
- Exposure to LEDs can result in retinal damage if the accessible emission limits are exceeded.

**Public health significance—Why is this work of significance for public health?**

- Children are known to be a sensitive population with respect to ocular hazards.
- This study investigates the eye safety of 103 LEDs integrated into a sample of 50 toys purchased during a 3-year period by a national market surveillance authority.

**Public health implications—What are the key implications or messages for practitioners, policy makers and/or researchers in public health?**

- In total, 30% of the tested toys had at least one LED that did not comply with the normative eye safety requirements: limits for the blue-light hazard and the retinal thermal hazard were exceeded by about 15% of the tested LEDs.
- Market surveillance should be reinforced to protect children’s eyes. The eye safety of LEDs in toys should be assessed at a viewing distance of 100 mm.

**Abstract:**

Between 2021 and 2023, the French market surveillance authority anonymously bought a sample of 50 toys containing a total of 103 individual LEDs from French retailers and had them tested by third-party laboratories accredited to perform tests in compliance with the international standard for electric toy safety IEC 62115, editions 2003 and 2017. Compliance with the harmonized EN IEC version of this standard is mandatory in the European Union under directive 2009/48/EC. The results revealed that 15 toys (30% of the tested toys) had at least one LED that did not comply with the eye safety requirements: 10 LEDs were non-compliant with respect to the retinal blue-light hazard, and 5 LEDs were non-compliant with respect to the retinal thermal hazard. A subsample of 10 toys with a total of 19 LEDs was assessed using the respective test methods corresponding to the 2003 and 2017 editions of the standard. The 2017 test method was found to be more permissive than the 2003 test method, a conclusion explained by the use of different risk assessment distances.

## 1. Introduction

Light-emitting diodes (LEDs) have become ubiquitous in toys, exhibiting a wide range of functions ranging from use as low-intensity indicators to employment as bright, multicolored lighting devices. The potential hazards associated with LEDs for children’s ocular health are garnering increasing attention because of their ability to reach high intensity levels. Beyond the context of toys, high-intensity LEDs have become prevalent in numerous objects that are readily accessible to children, including miniature torch lights and key chains.

The dominant ocular hazards associated with LEDs used in toys concern the retina. Photothermal injuries may happen when the incident optical power induces an unsafe temperature elevation in the retina. Photochemical injuries to retinal tissues can be triggered by short-wavelength photons in the spectral range spanning from the UV-A to the blue-and-green region of the optical spectrum [1,2].

The prevailing requirements for evaluating the optical safety of LEDs in toys are set out in the 2017 version of the IEC 62115 electric toy safety standard [3]. In the European Union, this standard was subsequently transposed into the 2020 European standard EN IEC 62115 and into 34 national standards, such as the NF EN IEC 62115 standard in France and the BS EN IEC 62115 standard in the UK. In the European Union, the limits of the EN IEC 62115 standard are considered mandatory. The IEC 62115:2017 standard was the first standard to introduce emission limits expressed in terms of luminous and radiant intensity values. The previous version of the same standard had been previously issued in 2003 by the IEC [4] and transposed as the EN IEC 62115:2005 standard. It dealt with the optical safety of LEDs by defining exposure limits expressed in terms of radiant flux and photochemical energy, an assessment based on an optical power measured at a given distance, within a given aperture, and over a given exposure time.

This study presents the results of a three-year campaign (2021–2023) carried out by the French market surveillance authority to assess the optical safety of LEDs found in a sample of 50 commercially available toys. This study also compares the results obtained with a subsample of 10 toys tested with two different test methods that were used by the chosen accredited test laboratories during the course of this study: the IEC 62115:2003 and IEC 62115:2017 test methods.

## 2. Materials and Methods

A total of 50 toys with LEDs were bought in different toy stores in France by the governmental market surveillance authority—the Directorate-General for Competition, Consumer Affairs, and Fraud Prevention (DGCCRF). In these toys, a total of 103 LEDs were selected for testing. The selected toys were clearly intended to be used by children under the age of 14 for the sole purpose of play. The sampling strategy was not based on an actual analysis of the toy market. Instead, the objective was to cover the main categories of common toys. The choice was based on the indisputable presence of LEDs that were clearly shown on the toy packages or well visible on models displayed on the store shelves.

These LEDs were tested for compliance with the IEC 62115 standard by three accredited test laboratories. The three laboratories are France’s national metrology and testing institute (LNE), accredited by the French accreditation committee COFRAC; TSU, accredited by the Slovak national accreditation service SNAS; and Intertek France, accredited by COFRAC. Intertek and TSU applied the IEC 62115:2003 test method, while LNE used the IEC 62115:2003 and IEC 62115:2017 test methods.

### 2.1. Toy Description

The tested toys all belonged to very common categories of commercially available toys: stuffed animals, robots, balls, spinning tops, fire trucks, helicopters, motorcycles, aircraft, yoyos, pocket lamps, pens, plastic guns, key chains, head diadems, night lights, etc. The complete list of the 50 tested toys is provided in a spreadsheet available in the Supplementary Materials. The total number of tested LEDs in this sample of toys is 103.

### 2.2. Optical Measurements and Eye Safety Requirements

In accordance with the test conditions listed in both versions of the IEC 62115 standards, all the tested LEDs and their associated circuitry were carefully extracted from the toys. During the optical measurements, the LEDs were powered by new batteries fitted in the original battery compartment of a given toy. In the case of LEDs used without any current-limiting components, stable experimental conditions could not be achieved. In this case, the LEDs were connected to a laboratory power supply operated at a controlled nominal voltage corresponding to the toy manufacturer’s specifications. Optical measurements of the extracted LEDs were performed in a dark enclosure without stray light.

The optical measurements were made according to the protocol of either IEC 62115:2003 or/and IEC 62115:2017, according to the choice made by the test laboratory. The 2003 test method is based on the requirements used to define Class 1 of laser safety, corresponding to conditions of safe viewing when the exposure is long and intentional. The radiant flux was measured through a circular aperture measuring 7 mm diameter, at four different distances between 14 mm and 100 mm [4]: two distances to assess the blue-light hazard, and two distances to assess the thermal hazard. For the blue-light hazard, the photochemical dose (unit: J) was determined by multiplying the blue-light weighted radiant flux by an exposure time of 100 s. To comply with the eye safety requirements, this dose must be less than an accessible limit value determined according to the emission wavelengths and the apparent angular dimensions of the LED. For the thermal hazard, the radiant flux is required to be less than an accessible limit value expressed in terms of optical power (unit: W) determined according to the emission wavelengths and the apparent angular dimensions of the LED.

In the 2017 edition of the safety standard [4], the optical quantity to assess is either the radiant intensity (unit: W/sr) or the luminous intensity (unit: cd). Either of these quantities can be measured. The LNE test laboratory chose to measure radiant intensity. Although intensity is not a function of viewing distance, the measurements were made at 200 mm, as recommended in the CIE standard method for measuring the intensity of an LED [5].

## 3. Experimental Results

The spreadsheet provided in the Supplementary Materials includes the detailed experimental results.

### 3.1. Overall Non-Compliance Rate

Out of the 50 tested toys, a total of 15 toys (30% of the tested toys) were found to be non-compliant with the IEC 62115 eye safety requirements, evaluated either with the 2003 or 2017 versions. These 15 toys each had at least one LED exceeding an emission limit concerning a retinal hazard (thermal hazard or blue-light hazard).

### 3.2. Influence of the Color of the Tested LEDs

Blue, green, amber and red LEDs were the most represented LEDs in the test sample. Such colored LEDs have a narrow spectral distribution in the visible spectral range. The tested white LEDs had either a wide spectral distribution corresponding to the common blue-pump phosphor-converted technology (pc-white) or a spectral distribution with three narrow peaks (blue, green and red) corresponding to an RGB technology (RGB-white). An infrared LED was found in a toy gun (this LED was intended to be used to “shoot” at a target fitted with a photodetector). This LED emitted invisible optical radiation at around 931 nm. Five LEDs were not identified in terms of spectral distribution (one of the three test laboratories did not report this information).

The number of non-compliant LEDs classified according to their color is shown in Table 1. There were 15 non-compliant LEDs in the tested sample of 103 LEDs (~15%). All the tested infrared, green, amber, and RGB white LEDs were compliant with the IEC 62115 requirements and met the eye safety requirements.

**Figure 1:**
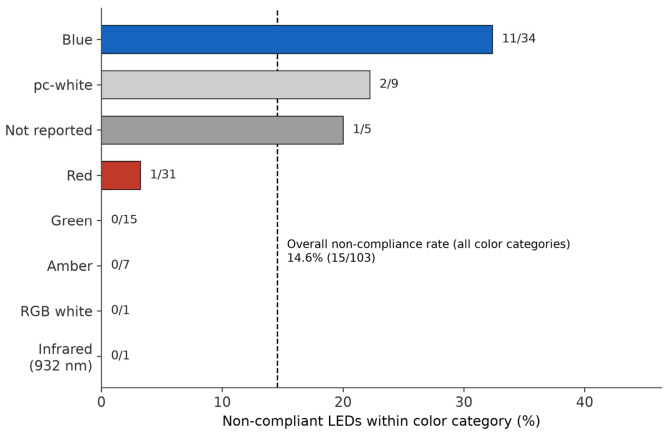
Non-compliance rate by LED color category. Data from Table 1 (*n* = 103 LEDs tested, 15 non-compliant).

Among the 15 non-compliant LEDs, there were 11 blue LEDs (accounting for 32% of the blue LEDs tested), representing a percentage of 73% of all the non-compliant LEDs. Two pc-white LEDs were non-compliant (accounting for 22% of the pc-white LEDs tested and 13% of the non-compliant LEDs tested), and so was one red LED (accounting for 3% of the red LEDs tested and 7% of the non-compliant LEDs). Figure 1 shows the distribution of non-compliant LEDs according to their colors.

### 3.3. Type of Retinal Hazard Identified in Non-Compliant LEDs

**Figure 2:**
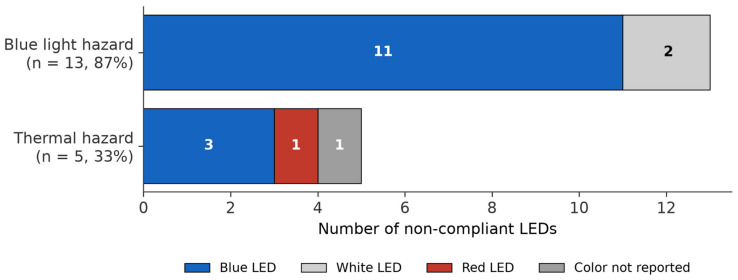
Retinal hazard types among the 15 non-compliant LEDs (data from Table 2).

The repartition of the different hazard types identified in non-compliant tested LEDs is shown in Table 2. Blue-light hazard constituted the type of retinal hazard that was most frequently identified as the source of non-compliance with eye safety requirements. This type was found for 87% of the non-compliant LEDs and applied to all the non-compliant blue LEDs and white LEDs. Emission limits concerning the thermal retinal hazard were exceeded by two blue LEDs and one red LED. A graphical summary of Table 2 is shown in Figure 2.

### 3.4. Comparison of the Assessment Results Obtained with the Two Different Test Methods

**Figure 3:**
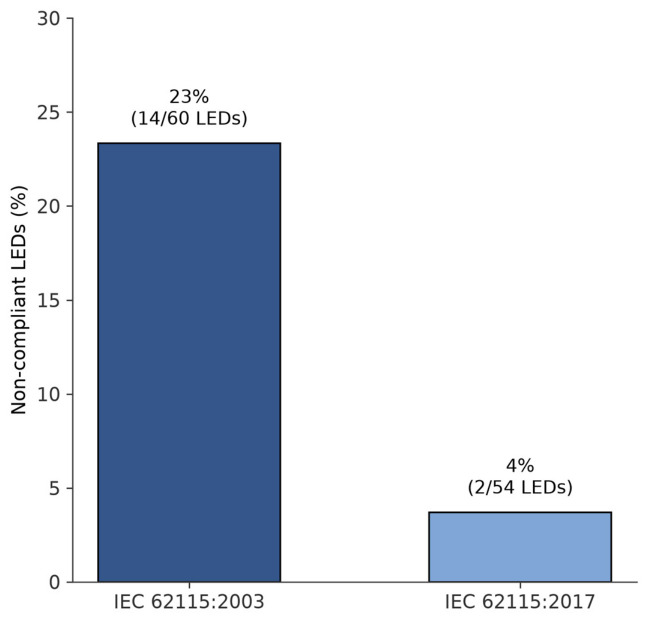
Non-compliance rate by test method. Each test method was applied to a different subset of LEDs. Ten LEDs were tested using both methods. One LED was found to be non-compliant with both test methods.

Table 3 and Figure 3 present the number of non-compliant LEDs identified in the 50 toys according to the test method applied by the accredited laboratories. Each test method was applied to approximately similar samples of LEDs, representing about one half of the 103 LEDs tested. However, the two test methods returned very different non-compliance percentages. The IEC 62115:2017 test method identified about six times fewer non-compliant LEDs than the IEC 62115:2003 test method.

Based on this result alone, it is not possible to conclude with certainty that the 2017 method is more permissive, because the methods were applied to different LEDs. To investigate the effect of the test method, a subsample of 10 toys (19 LEDs) was evaluated using both test methods. The results are detailed in the next section.

### 3.5. Comparison of the Assessment of 10 Toys Using the Two Different Test Methods

A subsample of 10 toys (identification numbers #41 to #50 in the Supplementary Materials spreadsheet) underwent testing using the respective methods corresponding to the IEC 62115 standards of 2003 and 2017. The total number of tested LEDs in this subsample is 19 (identification numbers #85 to #103).

Table 4 presents the detailed results regarding the LEDs tested in the subsample of 10 toys obtained with the IEC 62115:2003 and IEC 62115:2017 test methods.

Table 4 shows that 18 of the 19 tested LEDs complied with the requirements of the 2017 standard, but only 11 LEDs out of 19 were found to comply with the 2003 version. The seven LEDs that complied with the 2017 standard, but not with the 2003 standard, were blue and white LEDs.

**Figure 4:**
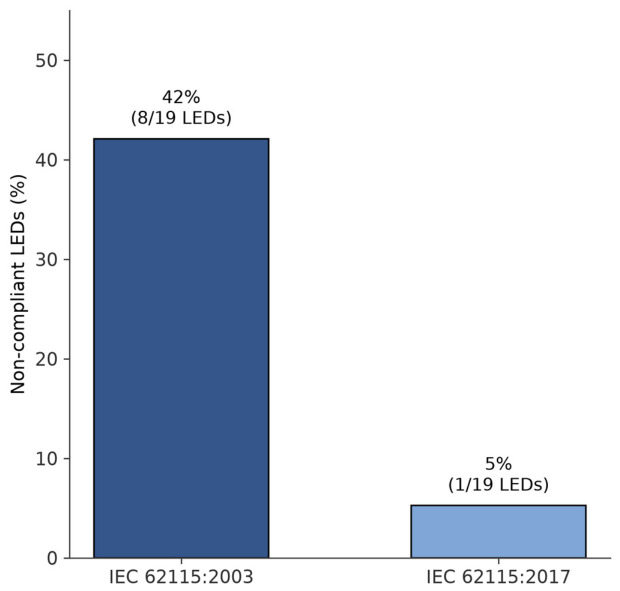
Non-compliance rate by test method. (Data from Table 5. Sample of 19 LEDs tested with both methods).

Table 5 and Figure 4 present the non-compliance percentage among the 10 toys, as assessed using both test methods. A total of eight toys (eight LEDs) did not comply with the 2003 standard, while only one toy (one LED) did not comply with the 2017 standard. This paired-method comparison supports the interpretation that the IEC 62115:2017 test method is more permissive than the IEC 62115:2003 test method. Table 5 shows that the 2017 test method identified seven times fewer non-compliant LEDs than the 2003 test method.

## 4. Discussion

The method introduced in the IEC 62115:2017 standard was first defined in a scientific paper [6], establishing accessible emission limits for LEDs in toys in terms of radiant and luminous intensity. Unlike the incident radiant flux at the eye used in the IEC 62115:2003 test method, intensities are not functions of viewing distance, and their values may be directly found in the LED datasheet, which simplifies risk assessment for toy manufacturers.

The emission limit values defined in [6] and used in the IEC 62115:2017 standard were based on a viewing distance of 200 mm. In comparison, the previous test method corresponding to IEC 62115:2003 used assessment distances of 100 mm down to 14 mm in some cases. As radiant flux is a function of the inverse square of viewing distance, doubling the assessment distance from 100 mm to 200 mm lowers the incident radiant flux by a factor of 4, thereby reducing the assessed risk level. The inverse-square law is indeed only valid for a point source. In this study, the tested LEDs all had a maximum diameter of 5 mm, while all the measurements were made at more than 100 mm. This distance is 20 times longer than the width of the light source, justifying the validity of the inverse-square law.

The difference between the measurement distances is the main cause of the discrepancy between the 2003 and 2017 test methods. A theoretical analysis of the intensity limits for LEDs used in toys also concluded that a greater measurement distance lowers the assessed risk level for the eye [7]. The choice of a greater assessment distance in the 2017 test method is questionable. In fact, children younger than two years of age have a shorter ocular focal length [7,8] and shorter arms than adults, suggesting that they are likely to manipulate their toys at short distances, typically less than the distance of 200 mm that was chosen to define the current normative intensity limits. Observational studies of child–device interactions suggest that viewing distances below 200 mm are common in early childhood [9].

The blue-light hazard poses a potentially greater risk to the eyes of children in comparison to those of adults. This phenomenon can be attributed to the combination of two factors. Firstly, the crystalline lens of a child exhibits a higher transparency to blue light, particularly before the age of 8 years [8]. Secondly, the geometric aperture of a child’s eye is up to three times greater than that of an adult’s eye [10]. Consequently, the same illuminance level at the eye generates higher retinal illuminance in the case of children. Modeling studies indicate that retinal exposure in children may be up to three times higher than in adults under identical external illumination conditions [10].

Exposure to high-intensity LEDs can result in transient visual impairment, characterized by instances of temporary loss of vision (saturation glare) and the persistence of afterimages within the visual field. This phenomenon is attributed to the photobleaching of retinal photoreceptors [11,12]. These effects are well documented in both psychophysical and physiological studies describing glare and visual performance degradation with high-luminance sources [13,14]. If the limit for the blue-light hazard is exceeded, there is a risk of acute retinal damage [11,15].

Importantly, even if the blue-light-hazard limit for acute exposure is not reached, repeated exposure might be associated with long-term damage. Indeed, although the exact role of repeated light exposure in age-related ocular diseases is difficult to establish, a recent study demonstrated that rats exposed to blue and white lights for 15 days at retinal doses that were safe in a single acute exposure displayed irreversible retinal damage [16]. Additional animal studies on commercial white and blue LEDs corroborate these findings and highlight oxidative-stress-mediated photoreceptor degeneration [17]. Complementary studies on retinal pigment epithelium cells support the notion that mechanisms involving reactive oxygen species, mitochondrial dysfunction, and lipofuscin-related phototoxicity under blue-light exposure are involved [18]. Increasing evidence is emerging from animal and cell studies, as well as from epidemiological studies, supporting the role of light exposure in the occurrence of retinal diseases [19,20,21,22]. Moreover, a recent study has shown that, in humans, evening light exposure exceeding 1000 lx is significantly associated with an increased risk of incident age-related macular degeneration (AMD), cataracts, and primary open-angle glaucoma. Significant time–response relationships were observed, with per-hour exposure to >2250 lx further elevating the risk of AMD and glaucoma [23]. This suggests that repeated exposure to artificial lights, early in life, may have long-term cumulative consequences for ocular health.

The risks of high-intensity LEDs could be mitigated by the aversion response in children. However, the aversive response may not be fully developed in young children, whose retinas are immature [24]. In the case of older children, the aversive response can be intentionally disregarded for the sake of play, as evidenced by recent case reports of macular injuries in children exposed to hand-held high-intensity LED devices [25,26] or laser pointers [27,28] during recreational activities. In addition, the perception of the brightness of LEDs emitting pure blue light is diminished due to the lower sensitivity of vision in this spectral range, even when radiant intensity is high [29]. In the absence of a high-brightness cue, the aversion response is significantly reduced or even absent [10,18]. This mismatch between perceived brightness and photochemical hazard remains a critical limitation of current safety approaches and of risk communication regarding blue-rich LED sources.

There are several limitations pertaining to this study. The selection process for the tested toys was based on the identification of toys containing LEDs by the DGCCRF controllers during their visits to toy stores. The selection was not based on market analysis. Our sample is relatively small in comparison with the number of available toys on the market. In addition, the sample is also geographically limited to France. However, the tested toys were not manufactured in France and bore multilingual text on their packages. These toys are likely to be sold on the global market and to be available in other countries. The possible variability between manufacturers of similar categories of toys could not be evaluated. It is therefore impossible to identify specific types of toys that would present a higher risk to a child’s eyes. Potential variability between the testing institutes was not evaluated. Nevertheless, selecting accredited laboratories was the best possible option to minimize this effect. The comparison between the two different IEC methodologies was limited to a subsample of 10 toys due to time and budget constraints. The observed differences are nonetheless supported by theoretical considerations about the risk assessment distance [7].

## 5. Conclusions

During a three-year period of governmental market surveillance conducted in France between 2021 and 2023, 50 toys with 103 LEDs were tested for their eye safety by three independent accredited laboratories. The measurements revealed that 30% of the tested toys had at least one LED that was not rated as being safe for the eye, potentially exposing children to a risk of retinal damage. The non-compliance instances mostly concerned blue LEDs, in addition to a few white and red LEDs. The main type of potential hazard identified was the retinal blue-light hazard. A lower number of blue, white and red LEDs also exhibited a potential risk of retinal thermal damage. These findings are highly relevant for surveillance but should not be interpreted as a statistically representative non-compliance estimate for the entire toy market. The three accredited test laboratories used two different test methods corresponding to successive editions of the same international safety standard, IEC 62115: the 2003 and 2017 editions. A subsample of 10 toys with a total of 19 individual LEDs was assessed using both methods. The 2017 test method was found to be more permissive than the 2003 test method, essentially because the viewing distance considered in the assessment method was extended from 100 mm and below (2003 edition) to 200 mm (2017 edition). Consequently, when using toys that are compliant with the current safety standard (2017), children may be exposing their eyes to higher risks than when using toys that were approved using the previous safety standard (2003).

Regulatory agencies and health authorities are currently working on the revision of the eye safety assessment for the electric toy safety standard to ensure adequate protection of children’s visual health.

## Figures and Tables

**Table 1:** Number of non-compliant LEDs according to their color.

Color	Number of LEDs Tested	Number of Non-Compliant LEDs	Percentage of Non-Compliant LEDs in the Color Category	Percentage of Non-Compliant LED with Respect to All Non-Compliant LEDs (15)	Reason for Non-Compliance
Blue	34	11	32%	73%	blue-light hazard (8 LEDs) thermal hazard and blue-light hazard (3 LED)
Green	15	0	0	0	-
Amber	7	0	0	0	-
Red	31	1	3%	7%	thermal hazard
pc-white	9	2	22%	13%	blue-light hazard
RGB white	1	0	0	0	-
Infrared 932 nm (not a color, invisible radiation)	1	0	0	0	-
Color not reported by test lab	5	1	20%	7%	thermal hazard
TOTAL	103	15	14.6%	100%	blue-light hazard or thermal hazard

**Table 2:** Repartition of the hazard types identified in the 15 non-compliant tested LEDs.

Retinal Hazard	Number of Non-Compliant LEDs	Color of Non-Compliant LEDs	Percentage
Blue-light hazard	13	11 blue LEDs	87%
		2 white LEDs	
Thermal hazard	5	3 blue LEDs	33%
		1 red LED	
		1 unknown type of LED	

Note: A total of 3 blue LEDs were non-compliant for both thermal and blue-light hazards. They were included in both categories of this Table. For this reason, the percentages do not add up to 100%.

**Table 3:** Repartition of the non-compliant LEDs identified in the 50 toys according to the test method used by the accredited test laboratories.

Test Method	Number of LEDs Tested	Non-Compliant LEDs	Non-Compliance Percentage
IEC 62115:2003	60	14	23%
IEC 62115:2017	54	2	4%

Note 1: Each test method was applied to a different subset of LEDs. Note 2: Ten LEDs were tested using both methods. Note 3: One LED was found to be non-compliant with both test methods.

**Table 4:** Experimental results of the assessment of 10 toys with the respective test methods corresponding to IEC 62115:2003 and IEC 62115:2017. The symbol “✓” on green background means that the given LED complies with the requirements of the given standard. The red background means that the given LED does not comply with the requirements of the given standard.

Toy/LED		Assessment Using IEC 62115:2003	Assessment Using IEC 62115:2017
Toy 41	Blue (#85)	**✓**	**✓**
Toy 42	Blue (#86)	**✓**	**✓**
	Green (#87)	**✓**	**✓**
	Red (#88)	**✓**	**✓**
Toy 43	Blue (#89)	**Blue-light hazard**	**✓**
Toy 44	Blue (#90)	**Blue-light hazard**	**✓**
	Green (#91)	**✓**	**✓**
	Red (#92)	**✓**	**✓**
Toy 45	Blue (#93)	**Blue-light Hazard**	**✓**
	Green (#94)	**✓**	**✓**
	Red (#95)	**✓**	**✓**
Toy 46	Blue (#96)	**Blue-light hazard** **Thermal hazard**	**✓**
	Red (#97)	**✓**	**✓**
Toy 47	Blue (#98)	**Blue-light hazard** **Thermal hazard**	**Blue-light hazard** **Thermal hazard**
	Red (#99)	**✓**	**✓**
Toy 48	White (#100)	**Blue-light hazard**	**✓**
Toy 49	White (#101)	**Blue-light hazard**	**✓**
Toy 50	Blue (#102)	**Blue-light hazard**	**✓**
	Amber (#103)	**✓**	**✓**

**Table 5:** Percentage of non-compliant LEDs among the 10 toys assessed using both test methods.

Tested LED	Test Method	Non-Compliant LEDs	Non-Compliance Percentage
19 LEDs, each tested with both methods	IEC 62115:2003	8 out of 19	42%
	IEC 62115:2017	1 out of 19	5%

## Data Availability

The original dataset presented in this study is included in the article/Supplementary Material. Further inquiries can be directed to the corresponding author.

## Supplementary Materials

The following supporting information can be downloaded at https://www.mdpi.com/article/10.3390/ijerph23070880/s1. Table S1: Measurement result database of the 50 tested toys (103 LEDs).
