# Supplementary material for: Eye Safety of Light-Emitting Diodes in Toys: The Results of Three Years of Market Surveillance
Source: Int J Environ Res Public Health. 2026 Jul 6;23(7):880. doi: 10.3390/ijerph23070880 (PMC13412091; doi:10.3390/ijerph23070880)
Supplement: Supplementary file 1 [file ijerph-23-00880-s001.zip › ijerph-4331808-supplementary.pdf]

Table S1. Measurement result database of the 50 tested toys (103 LEDs).

Year of purchase	Date of testing	Toy Id.	Toy description	LED Id.	LED color	IEC 62115 Version	LED peak [nm]	Radiant intensity [W/sr]	Limit [W/sr]	Ratio intensity/limit	Radiant flux [mW]	Limit [mW]	Ratio flux/ limit	Photochemical dose [mJ]	Limit [mJ]	Ratio dose/limit	Test Lab	Pass or Fail	Pass IEC 62115:2003 ?	Pass IEC 62115:2017 ?
2021	04/05/2021	1	Diadème lumineux à oreilles	1	Green	2003	526				0.0363	1.92	0.019	0.14	130	0.001	Intertek	PASS	YES	
2021	04/05/2021	1	Diadème lumineux à oreilles	2	Blue	2003	449				0.105	1.46	0.072	0.88	3.9	0.226	Intertek	PASS	YES	
2021	04/05/2021	1	Diadème lumineux à oreilles	3	Amber	2003	586				0.0424	1.78	0.024	0.68	2	0.340	Intertek	PASS	YES	
2021	04/05/2021	1	Diadème lumineux à oreilles	4	Blue	2003	466				0.216	0.7	0.309	4.6	8.1	0.568	Intertek	PASS	YES	
2021	04/05/2021	1	Diadème lumineux à oreilles	5	Red	2003	617				0.0602	1.46	0.041				Intertek	PASS	YES	
2021	20/05/2023	2	LED Balloon	6	Green	2003	539				4.72E-03	6.94	0.001	0.25	230	0.001	Intertek	PASS	YES	
2021	20/05/2023	2	LED Balloon	7	Blue	2003	453				6.27E-03	6.94	0.001	0.78	4.5	0.173	Intertek	PASS	YES	
2021	08/06/2022	3	Toupie	8	Green	2003	525				0.15	1.9	0.079	2.6	123	0.021	LNE	PASS	YES	
2021	08/06/2022	3	Toupie	9	Blue	2003	439				1.27	1.9	0.668	33.8	3.9	8.667	LNE	FAIL	NO	
2021	08/06/2022	3	Toupie	10	Red	2003	629				0.17	1.9	0.089	/	/	/	LNE	PASS	YES	
2021	08/06/2023	4	Magic circuit	11	Blue	2003	452				0.013	9.9	0.001	0.64	4.3	0.149	LNE	PASS	YES	
2021	08/06/2023	4	Magic circuit	12	Blue	2003	448				0.17	1.4	0.121	4.3	3.9	1.103	LNE	FAIL	NO	
2022	08/06/2023	4	Magic circuit	13	Red	2003	631				0.11	1.4	0.079	/	/	/	LNE	PASS	YES	
2021	08/06/2023	4	Magic circuit	14	Red	2003	632				0.004	9.9	0.000	/	/	/	LNE	PASS	YES	
2021	25/02/2021	5	Camion de pompier	15	Red	2003	633				45.5E-3	6.94	0.007			0.007	Intertek	PASS	YES	
2021	14/10/2021	6	Light ball	16	not reported	2003											TSU	PASS	YES	
2022	14/10/2021	7	Helicopter	17	not reported	2003											TSU	PASS	YES	
2021	03/11/2021	8	Zero gravity laser	18	not reported	2003											TSU	PASS	YES	
2021	24/11/2021	9	Advance shot blaster	19	not reported	2003					1.23	0.384	3.203				TSU	FAIL	NO	
2021	05/10/2021	10	Peluche poulpe réversible avec LED	20	Green	2003	518				0.04	1.2	0.033	1.1	/	0.030	LNE	PASS	YES	
2021	05/10/2021	10	Peluche poulpe réversible avec LED	21	Blue & ambe	2003	455/597				0.08	1.2	0.067	1.3	/	0.050	LNE	PASS	YES	
2021	05/10/2021	10	Peluche poulpe réversible avec LED	22	Blue & ambe	2003	455/605				0.18	1.2	0.150	3.5	/	0.450	LNE	PASS	YES	
2021	05/10/2021	10	Peluche poulpe réversible avec LED	23	Blue	2003	455				0.3	0.5	0.600	25	/	4.700	LNE	FAIL	NO	
2021	15/11/2023	11	Style and play unicorn	24	not reported	2003											TSU	PASS	YES	
2021	22/03/2021	12	Yoyo de compétition avec lumière	25	Red	2003	639				0.15	4.48	0.033			0.033	Intertek	PASS	YES	
2021	14/12/2021	13	Veilleuse de poche Frozen II	26	RGB	2003											LNE	PASS	YES	
2021	08/12/2021	14	Sucette laser pop super message fraise	27	Red	2003	626				0.47	9.88	0.048	/	/		LNE	PASS	YES	
2021	07/12/2021	15	Spray torch lemon 20 ml	28	White	2003	White				1.3	7.8	0.167	4.25	/	0.520	LNE	PASS	YES	
2021	23/03/2023	16	Balle lumineuse	29	Blue	2003	459				0.57	0.39	1.462	12	5.9	2.034	Intertek	FAIL	NO	
2021	23/03/2023	16	Balle lumineuse	30	Red	2003	631				0.28	0.39	0.718				Intertek	PASS	YES	
2021	26/03/2021	17	Yoyo lumineux	31	Red	2003	632				1.15	0.96	1.198				Intertek	FAIL	NO	
2021	14/12/2021	18	Ma corde à sauter lumineuse	32	Blue	2003	486				0.4	0.78	0.513	17	20.5	0.829	LNE	PASS	YES	
2021	14/12/2021	18	Ma corde à sauter lumineuse	33	Red	2003	618				0.12	0.78	0.154				LNE	PASS	YES	
2021	11/05/2023	19	Stylo top model	34	White	2003	White				0.04	6.7	0.006	0.42	/	0.040	LNE	PASS	YES	
2021	13/01/2023	20	Ballon gonflable à DEL	35	Red & blue	2003	Purple				0.09	2.85	0.032	0.76	/	0.120	LNE	PASS	YES	
2021	24/09/2021	21	Mon 1er appareil photo	36	Amber	2003	594				0.072	0.39	0.185	2.8	3000	0.001	Intertek	PASS	YES	
2021	16/09/2021	22	Piano rigolo elephant	37	Red	2003	629				0.0135	0.65	0.021				Intertek	PASS	YES	
2021	08/11/2021	23	Lumifete Esla	38	White	2003	White				0.17	2.2	0.077	1.83	/	0.260	LNE	PASS	YES	
2021	14/12/2021	24	Robot danseur	39	Amber	2003	592				0.01	0.75	0.013	0.27	2700	0.000	LNE	PASS	YES	
2021	14/12/2021	24	Robot danseur	40	Green	2003	524				0.01	0.78	0.013	0.42	118	0.004	LNE	PASS	YES	
2021	14/12/2021	24	Robot danseur	41	Blue	2003	466				0.01	9.88	0.001	0.3	8.1	0.037	LNE	PASS	YES	
2021	14/12/2021	24	Robot danseur	42	Blue	2003	458				0.01	6.66	0.002	0.32	5.6	0.057	LNE	PASS	YES	
2021	14/12/2021	24	Robot danseur	43	Blue	2003	467				0.01	0.78	0.013	0.64	8.5	0.075	LNE	PASS	YES	
2021	14/12/2021	24	Robot danseur	44	Red	2003	622				0.01	6.66	0.002				LNE	PASS	YES	
2022	14/12/2021	24	Robot danseur	45	Red	2003	624				0.01	0.78	0.013				LNE	PASS	YES	
2022	16/12/2021	25	Command pursuit challenge	46	Amber	2003	587				0.05	0.78	0.064	2.6	2140	0.001	LNE	PASS	YES	
2022	16/12/2021	25	Command pursuit challenge	47	Red	2003	626				0.02	9.88	0.002				LNE	PASS	YES	
2022	16/12/2021	25	Command pursuit challenge	48	Infrared	2003	932				3.38	5.53	0.611				LNE	PASS	YES	
2022	28/07/2021	26	Animal unicorn and dog	49	Amber	2003	594				0.052	4.48	0.012	0.2	3000	0.000	Intertek	PASS	YES	
2022	05/09/2022	27	Mitraillette noire	50	Green	2017	523	0.0043	0.75	0.006							LNE	PASS		YES
2022	05/09/2022	27	Mitraillette noire	51	Red	2017	629	0.0065	0.76	0.009							LNE	PASS		YES
2022	05/09/2022	27	Mitraillette noire	52	Red	2017	632	0.0074	0.76	0.010							LNE	PASS		YES
2022	05/09/2022	27	Mitraillette noire	53	Green	2017	523	0.012	0.75	0.016							LNE	PASS		YES
2022	05/09/2022	27	Mitraillette noire	54	Blue	2017	459	0.012	0.05	0.240							LNE	PASS		YES
2022	05/09/2022	27	Mitraillette noire	55	Blue	2017	460	0.022	0.05	0.440							LNE	PASS		YES
2022	05/09/2022	28	Arche musical hibou	56	Red	2017	642	0.0032	0.76	0.004							LNE	PASS		YES
2022	05/09/2023	29	Veilleuse koala	57	Blue	2017	445	0.1	0.13	0.769							LNE	PASS		YES
2022	24/10/2022	30	Crystal ball molle	58	Red	2017	626	0.028	0.76	0.037							LNE	PASS		YES
2022	24/10/2022	30	Crystal ball molle	59	Green	2017	535	0.052	0.76	0.068							LNE	PASS		YES
2022	24/10/2022	30	Crystal ball molle	60	Blue	2017	460	0.118	0.05	2.360							LNE	FAIL		NO
2022	13/09/2022	31	Ballons illuminés	61	Green	2017	521	0.012	0.67	0.018							LNE	PASS		YES
2022	13/09/2022	31	Ballons illuminés	62	Red	2017	636	0.018	0.76	0.024							LNE	PASS		YES
2022	13/09/2022	31	Ballons illuminés	63	Blue	2017	451	0.005	0.05	0.100							LNE	PASS		YES
2022	13/09/2022	31	Ballons illuminés	64	Blue	2017	465	0.009	0.06	0.150							LNE	PASS		YES
2022	13/09/2022	31	Ballons illuminés	65	Blue	2017	452	0.04	0.13	0.308							LNE	PASS		YES
2022	13/09/2022	32	Toupie gyroscopique	66	Red	2017	627	0.006	0.76	0.008							LNE	PASS		YES
2022	13/09/2022	32	Toupie gyroscopique	67	Green	2017	513	0.012	0.43	0.028							LNE	PASS		YES
2022	13/09/2022	32	Toupie gyroscopique	68	Blue	2017	466	0.016	0.06	0.267							LNE	PASS		YES
2022	09/11/2022	33	Flashing Spiky ball	69	Red	2017	633	0.0026	0.76	0.003							LNE	PASS		YES
2022	09/11/2022	33	Flashing Spiky ball	70	Blue	2017	467	0.0006	0.07	0.009							LNE	PASS		YES
2022	13/01/2023	34	Stylo boxeur halloween	71	Red	2017	634	0.018	0.76	0.024							LNE	PASS		YES
2022	13/01/2023	35	Sucette bonbon lumineuse	72	Red	2017	635	0.023	0.76	0.030							LNE	PASS		YES
2022	13/01/2023	36	Porte-clé Pieuvre verte	73	White	2017	White	0.091	0.13	0.700							LNE	PASS		YES
2022	17/03/2023	37	Ballons lumineux	74	Red	2017	618	0.00083	0.76	0.001							LNE	PASS		YES
2022	17/03/2023	37	Ballons lumineux	75	Green	2017	513	0.00118	0.18	0.007							LNE	PASS		YES
2022	17/03/2023	37	Ballons lumineux	76	White	2017	White	0.00105	0.13	0.008							LNE	PASS		YES
2022	17/03/2023	37	Ballons lumineux	77	Blue	2017	458	0.00097	0.05	0.019							LNE	PASS		YES
2022	17/03/2023	37	Ballons lumineux	78	Blue	2017	461	0.00176	0.05	0.035							LNE	PASS		YES
2022	17/03/2023	38	Feu stop gris zeeman	79	White	2017	White	0.0084	0.13	0.065							LNE	PASS		YES
2021	17/03/2023	38	Feu stop gris zeeman	80	White	2017	white	0.0093	0.13	0.072							LNE	PASS		YES
2021	17/03/2023	39	L'intrépide moto	81	Amber	2017	588	0.00014	0.76	0.000							LNE	PASS		YES
2021	17/03/2023	40	Veilleuse musicale bébé ours	82	Red	2017	628	0.00074	0.76	0.001							LNE	PASS		YES
2021	17/03/2023	40	Veilleuse musicale bébé ours	83	Green	2017	515	0.00067	0.57	0.001							LNE	PASS		YES
2021	17/03/2023	40	Veilleuse musicale bébé ours	84	Blue	2017	452	0.0084	0.05	0.168							LNE	PASS		YES
2023	21/02/2024	41	Toupie Quad Strike	85	Blue	both	411	0.0091	0.08	0.114	0.071	9.9	0.007	3.5	3.9	0.897	LNE	PASS	YES	YES
2023	21/02/2024	42	Submarine Projector	86	Blue	both	464	0.0153	0.06	0.255	0.056	3.4	0.016	5.6	7.4	0.757	LNE	PASS	YES	YES
2023	21/02/2024	42	Submarine Projector	87	Green	both	513	0.0077	0.43	0.018	0.029	3.4	0.009	2.9	71	0.041	LNE	PASS	YES	YES
2023	21/02/2024	42	Submarine Projector	88	Red	both	633	0.0062	0.76	0.008	0.024	3.4	0.007				LNE	PASS	YES	YES
2023	21/02/2024	43	Aircraft with light and sound	89	Blue	both	463	0.034	0.06	0.567	0.18	9.9	0.018	10.4	7.1	1.465	LNE	FAIL	NO	YES
2023	21/02/2024	44	Hélicoptère lumineux LED	90	Blue	both	452	0.03	0.05	0.600	1.44	1.9	0.758	18.2	4.8	3.792	LNE	FAIL	NO	YES
2023	21/02/2024	44	Hélicoptère lumineux LED	91	Green	both	519	0.025	0.6	0.042	1.22	1.9	0.642	17.3	115.3	0.150	LNE	PASS	YES	YES
2023	21/02/2024	44	Hélicoptère lumineux LED	92	Red	both	641	0.016	0.76	0.021	0.39	1.9	0.205				LNE	PASS	YES	YES
2023	21/02/2024	45	Pieuvre veilleuse peluche	93	Blue	both	450	0.0084	0.05	0.168	0.044	1.9	0.023	7.8	3.9	2.000	LNE	FAIL	NO	YES
2023	21/02/2024	45	Pieuvre veilleuse peluche	94	Green	both	515	0.004	0.43	0.009	0.022	1.9	0.012	0.43	77.8	0.006	LNE	PASS	YES	YES
2023	21/02/2024	45	Pieuvre veilleuse peluche	95	Red	both	627	0.002	0.76	0.003	0.066	1.9	0.035				LNE	PASS	YES	YES
2023	21/02/2024	46	Citrouille lumineuse	96	Blue	both	446	0.0085	0.05	0.170	0.92	0.78	1.179	40.6	3.9	10.410	LNE	FAIL	NO	YES
2023	21/02/2024	46	Citrouille lumineuse	97	Red	both	632	0.0039	0.76	0.005	0.43	0.78	0.551				LNE	PASS	YES	YES
2023	21/02/2024	47	Balle 70 mm météorite	98	Blue	both	460	0.079	0.05	1.580	4.8	1.53	3.137	177	7.1	24.930	LNE	FAIL	NO	NO
2023	21/02/2024	47	Balle 70 mm météorite	99	Red	both	631	0.014	0.76	0.018	1.19	1.53	0.778					PASS	YES	YES
2023	21/02/2024	48	Porte clé LED camion VW	100	White	both	White	0.043	0.13	0.331	0.805	var	0.110	21.7	var	1.810	LNE	FAIL	NO	YES
2023	21/02/2024	49	Porte clé lumineux et musical licorne	101	White	both	White	0.05	0.13	0.385	0.662	var	0.099	20	var	1.840	LNE	FAIL	NO	YES
2023	21/02/2024	50	Veilleuse dauphin	102	Blue	both	466	0.0034	0.06	0.057	0.28	0.78	0.359	13.5	8.1	1.667	LNE	FAIL	NO	YES
2023	21/02/2024	50	Veilleuse dauphin	103	Amber	both	591	0.0021	0.76	0.003	0.15	0.78	0.192	8.9	2580	0.003	LNE	PASS	YES	YES
